# Increased level of free‐circulating MtDNA in maintenance hemodialysis patients: Possible role in systemic inflammation

**DOI:** 10.1002/jcla.24558

**Published:** 2022-06-16

**Authors:** Xiao‐Yi Zhong, Yi Guo, Zhen Fan

**Affiliations:** ^1^ Department of Nephrology Clinical Medical College and The First Affiliated Hospital of Chengdu Medical College Chengdu China; ^2^ Department of Neurology, Sichuan Provincial People's Hospital University of Electronic Science and Technology of China Chengdu China; ^3^ Department of Geriatrics, Sichuan Provincial People's Hospital University of Electronic Science and Technology of China Chengdu China

**Keywords:** anuria, inflammation, maintenance hemodialysis, mitochondrial damage, mitochondrial DNA

## Abstract

**Background:**

Mitochondrial DNA (MtDNA) exposed to the extracellular space due to cell death and stress has immunostimulatory properties. However, the clinical significance of circulating MtDNA in maintenance hemodialysis (MHD) patients and the precise mechanism of its emergence have yet to be investigated.

**Methods:**

This cross‐sectional study consisted of 52 MHD patients and 32 age‐ and sex‐matched healthy controls. MHD patients were further categorized into high and low circulating cell‐free MtDNA (ccf‐MtDNA) groups based on the median value. Copy number of MtDNA was quantified using TaqMan‐based qPCR. Plasma cytokines were measured using ELISA kits. Reactive oxygen species (ROS) and mitochondrial membrane potential (Δψm) in peripheral blood mononuclear cells (PBMCs) were detected using DCFH‐DA or JC‐1 staining.

**Results:**

The copy numbers of ccf‐MtDNA in patients with MHD were higher than those in healthy controls, and these alterations were correlated with changes of cytokines TNF‐α and IL‐6. Adjusted model in multivariate analysis showed that the presence of anuria and longer dialysis vintage were independently associated with higher levels of ccf‐MtDNA. Meanwhile, although not statistically significant, an inverse correlative trend between urinary MtDNA and ccf‐MtDNA was observed in patients with residual urine. Afterward, using PBMCs as surrogates for mitochondria‐rich cells, we found that patients in the high ccf‐MtDNA group exhibited a significantly higher ROS production and lower Δψm in cells.

**Conclusions:**

Our data suggested that changes in ccf‐MtDNA correlate with the degree of inflammatory status in MHD patients, and that the excessive MtDNA may be caused by mitochondrial dysfunction and reduced urinary MtDNA excretion.

## Graphical abstract

The “out of place” MtDNA may be involved in the sterile inflammatory response in MHD patients. A longer dialysis vintage and the presence of anuria predict higher ccf‐MtDNA levels in MHD patients. Kidney may be partly responsible for MtDNA excretion. Mitochondrial damage likely has a contribution to the MtDNA leakage.

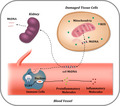

## 1 INTRODUCTION

Progressive decline in renal function leads to chronic kidney disease (CKD) and, ultimately, end‐stage renal disease (ESRD).^1^ Patients at this stage require renal replacement therapy, which is usually hemodialysis, to survive. Hemodialysis patients usually suffer from high cardiovascular morbidity and mortality due to chronic systemic inflammation.^2^ Nevertheless, in many cases, plasma inflammatory cytokines increase with disease progression independent of antigenic stimulation, such as bacterrial DNA fragments.^3^ In fact, inflammation not only occurs in response to pathogens but can also be induced without active infection. This so‐called “sterile inflammation” may be triggered by damage‐associated molecular patterns (DAMPs) derived from tissue injury and cell breakage.

Mitochondria play a central role in metabolism and are unique organelles that carry their own genome (mitochondrial DNA, MtDNA).^4^ According to the endosymbiotic theory, mitochondria may have originated from energy‐producing bacteria. Thus, most of MtDNA contains inflammatogenic unmethylated CpG motifs similar to those in bacterial DNA.^5^ In this regard, if MtDNA is released out of the cell and becomes extracellular MtDNA, it may trigger an inflammatory response by binding to pattern recognition receptors (PRRs) that typically recognize DNA from bacterial pathogen.^6^ Recent research has implicated MtDNA as a DAMP and marked increase in extracellular MtDNA was already found in different pathological disorders, such as trauma, sepsis, aging, caner, and immune‐mediated disease, which are characterized by a chronic inflammatory status.^7^, ^8^, ^9^, ^10^, ^11^ Likewise, an increased free‐circulating MtDNA has also been reported in patients with kidney disease.^12^, ^13^ However, the role of circulating cell‐free MtDNA (ccf‐MtDNA) in the process of chronic inflammation in MHD patients and the precise mechanism of its emergence remain to be defined.

Abnormal mitochondrial structure has been demonstrated in muscle, heart, liver, lung, endothelial cells, and monocytes under uremic conditions.^14^, ^15^, ^16^, ^17^, ^18^, ^19^ According to previous research, it is not surprising that upon mitochondrial damage, its DAMP content can be easily released into the extracellular space, which has been proven to be a trigger for inflammatory response and oxidative injury.^20^ Peripheral blood mononuclear cells (PBMCs) are abundantly rich in mitochondria. Recently, tests of PBMCs have been proposed to offer valid information about “general” mitochondrial health.^21^, ^22^, ^23^ Hence, we chose PBMCs in lieu of tissue biopsy collection to assess the integrity of mitochondria and determine the relationship between ccf‐MtDNA and mitochondria.

In the current study, we first sought to determine the association between ccf‐MtDNA and inflammatory cytokines in MHD patients. Furthermore, we attempted to decipher the potential mechanisms affecting its levels. These findings provide novel mechanistic insights into the linkage between released MtDNA and inflammation, and enable the identification of new therapeutic targets for this disease.

## 2 MATERIALS AND METHODS

### 2.1 Participants

A cross‐sectional study design was conducted in this research. All subjects were recruited from First Affiliated Hospital of Chengdu Medical College between January 2021 and May 2021. Inclusion criteria for the MHD group were as follows: (1) age above 40 and (2) patients undergoing regular hemodialysis prescription, three times a week, at least 6 months. The age‐ and sex‐matched healthy control (HC) group included donors who attended routine health examinations at the same hospital; these patients had no history or clinical evidence of any renal diseases. The exclusion criteria for all subjects were as follows: (1) active inflammatory diseases within the last 3 months; (2) malignant tumors; (3) immune system diseases; (4) active liver disease; and (5) acute cardiovascular and cerebrovascular disease. Finally, a total of 52 MHD patients and 32 healthy controls were enrolled in our examination program. The study protocol was approved by the Ethics Committee of First Affiliated Hospital of Chengdu Medical College and adhered to the principles outlined in the Declaration of Helsinki. All participants gave informed written consent.

### 2.2 Sampling strategy

Sample size was determined prior to data collection by a power analysis (G*Power, Version 3.0). According to the previous research,^13^ group sample sizes of 29 (MHD) and 29 (HC) achieved 95% power to reject the null hypothesis when the ccf‐MtDNA mean difference was 86.4 with standard deviations of 101.3 for the MHD group and 8.5 for the HC group, with a significance level (alpha) of 0.01 using a two‐sided two‐sample unequal‐variance *t* test. Power analyses indicated that the sample size in this study was appropriate

### 2.3 Blood and urine sampling

Blood samples from hemodialysis patients were obtained immediately prior to dialysis. Simultaneously, urine samples were collected. Samples were separated into cellular and cell‐free fractions within 2 h of the samples being drawn.

### 2.4 Blood biochemical parameters

Blood biochemical examinations, including blood creatinine (Cr), total cholesterol (TC), triglyceride (TG), low‐density lipoprotein cholesterol (LDL‐C), fasting blood sugar (FBS), albumin (Ab), hemoglobin (Hb), and vitamin D (VitD), were measured in clinical laboratories of the participating hospitals.

### 2.5 Measurement of MtDNA copy number using TaqMan qPCR

Cell‐free DNA was extracted from blood and urine samples using the QIAamp DNA Blood Mini Kit (Qiagen, Germantown, MD, USA) following the protocol of manufacturer. The MtDNA copy number was quantified by amplification of a highly conserved region of mitochondrial cytochrome b (Cytb) gene. The PCR primers and TaqMan probes were designed and synthesized by TsingKe Biological Technology (TsingKe Biotech, Beijing, China). Cytb: sense primer, 5′‐CGCTACCTTCACGCCAATG‐3′, antisense primer, 5′‐CGATGTGTAGGAAGAGGCAGATAA‐3′, FAM‐labeled TAMRA quenched probes, 5′‐CGCCTCAATATTC‐3′. The linearity of the quantitative assay was assessed using the template cloned into plasmid DNA and serially diluted to prepare a series of calibrators with known concentrations. Then, the absolute values of the MtDNA were determined by calculation from this standard curve, as previously described.^24^ Results were presented as MtDNA*×*10^5^ copies per μl.

### 2.6 Cytokine measurements

The plasma levels of tumor necrosis factor‐alpha (TNF‐α), interleukin‐1 beta (IL‐1β), and interleukin‐6 (IL‐6) were measured with the respective human TNF‐α (cat. no. PT518), IL‐1β (cat. no. PI305), and IL‐6 (cat. no. PI330) ELISA kits (Beyotime, Beijing, China) according to the manufacturer's protocols.

### 2.7 Measurement of reactive oxygen species (ROS) production and mitochondrial membrane potential (Δψm) by flow cytometry

PBMCs were isolated from whole blood using the Ficoll–Hypaque density gradient separation technique, and then, the PBMCs were suspended in PBS at a final concentration of ~105 cells/ml for flow cytometry. Cellular ROS production was determined with 2,7‐dichlorodihydrofluorescein diacetate (DCFH‐DA) (Beyotime, Beijing, China). JC‐1 dye (5,5′,6,6′‐tetrachloro‐1,1′,3,3′‐tetraethyl‐imidacarbocyanine iodide) (Beyotime, Beijing, China) was used for Δψm assessment. JC‐1 forms aggregates within healthy mitochondria that exhibit red fluorescence (emission, 590 nm) in polarized Δψm. In cells with altered mitochondrial function, JC‐1 only forms monomers exhibiting green fluorescence (emission, 527 nm) in the cytoplasm in depolarized Δψm. The changes in Δψm were recorded by flow cytometry for the determination of cells with green fluorescence. All staining was performed following the manufacturer's instructions and was analyzed using fluorescence‐activated cell sorting (FACS) software.

### 2.8 Statistical analyses

Data are expressed as means ± standard deviation (SD) or medium (25th and 75th percentiles) for continuous variables. The distribution of the data was tested using the Kolmogorov–Smirnov test. Normally distributed data were analyzed using an independent *t* test. Nonnormally distributed data were analyzed using the Mann–Whitney test. Spearman's tests were applied to determine the associations between continuous variables. Categorical data between two groups were compared using the chi‐square test (χ^2^ test) with Fisher's exact test. Logistic regression was used to describe and explain the relationship between dependent binary variables and independent variables. Values of *p* < 0.05 were considered statistically significant. All statistical analyses were conducted using SPSS 18.0.

## 3 RESULTS

### 3.1 Participant characteristics

The study comprised 52 subjects with ESRD undergoing hemodialysis therapy thrice a week and 32 normal healthy controls. Groups were matched for age and sex. Baseline clinical characteristics and laboratory data of the study population are summarized in Table 1. Of the included MHD patients, 55.8% were male, and the mean age was 51.59 ± 11.15 years. Glomerulonephritis (54.1%) was the most common primary cause of ESRD, followed by diabetic nephropathy (21.1%), hypertensive nephropathy (11.5%), and nephrosclerosis (5.7%). The mean dialysis vintage was 3.95 ± 1.74 years, and a total of 38 (73.0%) patients presented with anuria. There was a statistically significant difference between the two groups in terms of Cr, SBP, FBS, Ab, Hb, and VitD (all *p* < 0.05).

**TABLE 1:** Characteristics of the MHD and healthy control groups

Variable	HC (*n* = 32)	MHD (*n* = 52)	*t*/χ^2^/Z	*p*
Age (years)	49.78 ± 9.09	51.59 ± 11.15	−0.775	0.441
Male (*n*, %)	18 (56.2%)	29 (55.8%)	0.002	0.966
Cause of ESRD (*n*, %)				
Glomerulonephritis	NR	18 (34.6%)		
Diabetes mellitus	NR	11 (21.1%)		
Hypertension	NR	6 (11.5%)		
Nephrosclerosis	NR	3 (5.7%)		
Others	NR	14 (26.9%)		
Dialysis vintage (years)	NR	3.95 ± 1.74		
Anuria (*n*, %)	NR	38 (73.1%)		
Cr (μmol/L)	77.56 ± 16.42	888.16 ± 388.34	−15.030	<0.05
SBP (mmHg)	132.63 ± 16.50	149.21 ± 36.58	−2.832	<0.05
DBP (mmHg)	82.04 ± 12.15	89.21 ± 27.89	−1.618	0.173
TC (mmol/L)	3.90 ± 1.05	3.77 ± 1.64	0.396	0.692
TG (mmol/L)	1.65 ± 0.73	2.11 ± 1.60	−1.747	0.084
LDL‐C (mmol/L)	2.82 ± 1.11	3.04 ± 1.36	−0.781	0.437
FBS (mmol/L)	5.97 ± 1.18	7.64 ± 3.99	−2.296	<0.05
Ab (g/L)	46.83 ± 7.15	26.71 ± 9.45	10.347	<0.05
Hb (g/L)	142.09 ± 18.28	88.09 ± 25.60	10.399	<0.05
VitD (ng/ml)	34.12 ± 9.33	27.82 ± 9.14	3.209	<0.05
ccf‐MtDNA	1.78 (1.03, 3.10)	3.54 (2.56, 5.05)	−4.360	<0.05

Abbreviations: Ab, albumin (g/L); ccf‐MtDNA, circulating cell‐free mitochondrial DNA (10^5^ × 10^5^ copies/μl); Cr, creatinine (μmol/L); DBP, diastolic blood pressure (mmHg); FBS, fasting blood sugar (mmol/L); Hb, hemoglobin (g/L); LDL‐C, low‐density lipoprotein cholesterol (mmol/L); NR, not recorded; SBP, systolic blood pressure (mmHg); TC, total cholesterol (mmol/L); TG, triglyceride (mmol/L); VitD, vitamin D (ng/ml).

### 3.2 Level of ccf‐MtDNA and its relationship with cytokines in MHD patients

**FIGURE 1:**
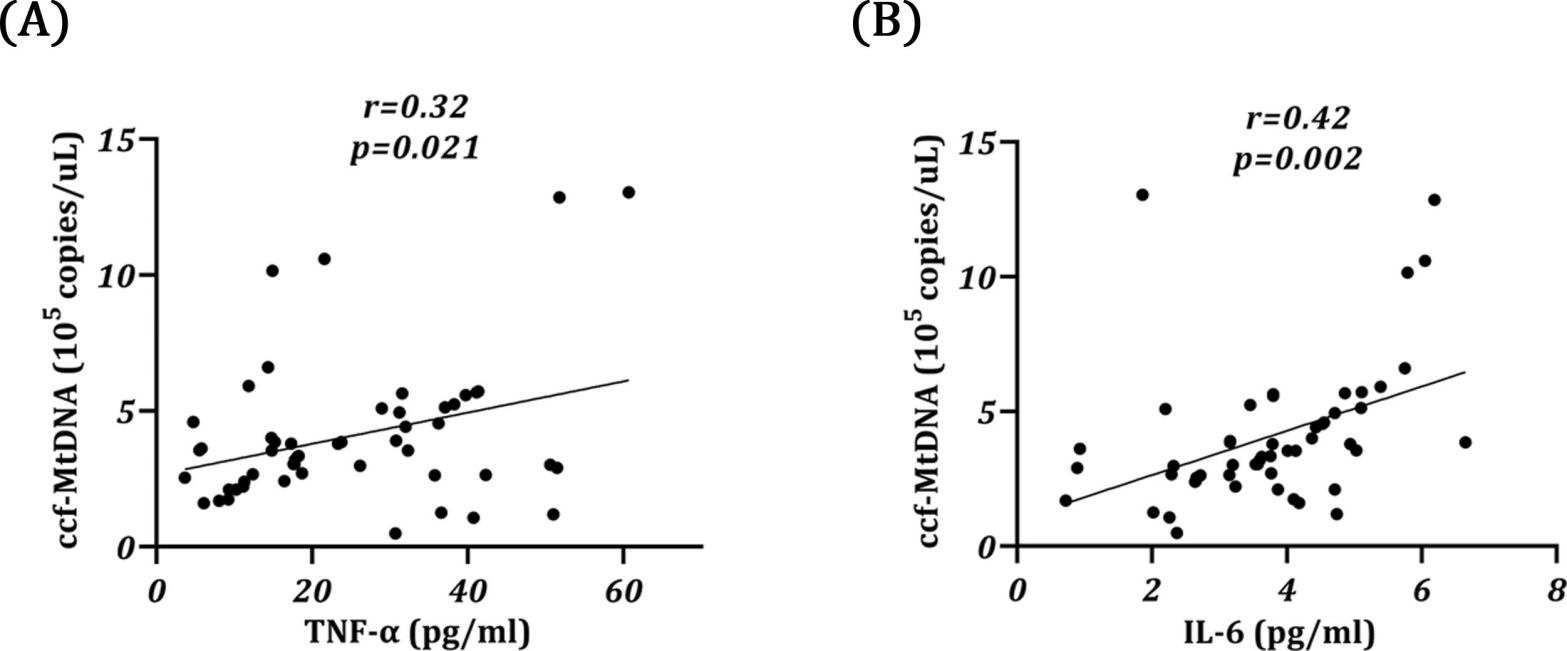
Correlation analysis between plasma TNF‐α/IL‐6 and ccf‐MtDNA in MHD patients. The results are represented as scatter plots, where each dot represents data obtained from one subject sample. Spearman correlation, r: Correlation coefficient. *n* = 52

Free‐circulating MtDNA was extracted from the plasma of all subjects. Cytb was applied to evaluate the quantity of MtDNA. As shown in Table 1, the content of ccf‐MtDNA in MHD patients was significantly higher than that in healthy controls (*p* < 0.05). Then, to evaluate systemic inflammation, the levels of circulating pro‐inflammatory cytokines TNF‐α, IL‐1*β*, and IL‐6 in the plasma were measured (Table 2). Among these cytokines, TNF‐α (*p* = 0.002) and IL‐6 (*p* < 0.001) levels were significantly higher in MHD groups than controls, whereas no significant difference was observed in IL‐1β. Spearman's rank correlation analysis showed that levels of ccf‐MtDNA in MHD patients were positively correlated with TNF‐α and IL‐6 (Figure 1; *r* = 0.32, *p* = 0.021 and *r* = 0.422, *p* = 0.002, respectively), suggesting that released MtDNA may be involved in the sterile inflammatory response of MHD.

**TABLE 2:** Plasma cytokine levels among the groups

Variable	HC (*n* = 32)	MHD (*n* = 52)	t/Z	*p*
TNF‐α (pg/ml)	14.5 (11.71, 18.03)	20.13 (12.87, 36.51)	−3.058	0.002
IL‐1*β* (pg/ml)	5.65 ± 1.18	6.15 ± 2.68	−1.152	0.253
IL‐6 (pg/ml)	1.53 ± 0.68	3.73 ± 1.35	−9.893	<0.001

### 3.3 Factors affecting ccf‐MtDNA levels in MHD patients

To identify factors associated with the high ccf‐MtDNA content, MHD patients were divided into two groups, low and high ccf‐MtDNA, using the median ccf‐MtDNA level as the cutoff value (low, <3.54 × 10^5^ copies/μl, *n* = 23; high, ≥3.54 × 10^5^ copies/μl, *n* = 23). Demographics and laboratory variables are given in Table 3. In univariate analysis, dialysis vintage (*p* = 0.008), anuria (*p* = 0.029), and VitD (*p* = 0.039) differed significantly across groups (Table 3). Subsequently, significant variables from the univariate analysis were subjected to the multivariate logistic regression analysis. Overall, dialysis vintage (OR = 1.62, *p* = 0.021) and anuria (OR = 6.05, *p* = 0.025) were independently associated with higher levels of ccf‐MtDNA after adjusting for the aforementioned confouding factors (Table 4).

**TABLE 3:** Baseline characteristics of MHD patients grouped by the median value of ccf‐MtDNA

Variable	Low ccf‐MtDNA (*n* = 23)	High ccf‐MtDNA (*n* = 23)	*t*/χ^2^/Z	*p*
Age (years)	52.57 ± 11.56	50.61 ± 10.87	0.630	0.531
Male (*n*, %)	14 (53.8%)	15 (57.6%)	0.078	0.780
Glomerulonephritis (*n*, %)	10 (43.4%)	8 (34.7%)	0.365	0.546
Dialysis vintage (years)	3.32 ± 1.82	4.58 ± 1.44	−2.757	0.008
Anuria (*n*, %)	3 (11.5%)	11 (42.3%)	4.78	0.029
Cr (μmol/L)	865.83 ± 273.48	910.50 ± 481.48	−0.411	0.683
SBP (mmHg)	147.93 ± 34.26	150.50 ± 39.41	−0.251	0.803
DBP (mmHg)	87.56 ± 29.71	90.85 ± 26.43	−0.423	0.674
TC (mmol/L)	3.82 ± 1.755	3.72 ± 1.56	0.218	0.828
TG (mmol/L)	1.97 ± 1.14	2.23 ± 1.97	−0.582	0.564
LDL‐C (mmol/L)	3.15 ± 1.41	2.93 ± 1.33	0.576	0.567
FBS (mmol/L)	8.42 ± 4.32	6.86 ± 3.55	1.424	0.161
Ab (g/L)	24.82 ± 8.11	28.60 ± 10.44	−1.456	0.152
Hb (g/L)	86.64 ± 29.90	89.54 ± 20.94	−0.406	0.687
VitD (ng/ml)	30.42 ± 8.59	25.23 ± 9.07	2.12	0.039

**TABLE 4:** Logistic regression analysis results of the association between ccf‐MtDNA content and clinical characteristics in MHD patients

Variable	Univariate analysis		Multivariate analysis	
	OR (95% CI)	*p*‐value	OR (95% CI)	*p*‐value
Dialysis vintage	1.60 (1.10–2.33)	0.013	1.62 (1.07–2.45)	0.021
Anuria	5.62 (1.34–23.56)	0.018	6.05 (1.26–29.09)	0.025
VitD	0.93 (0.87–1.00)	0.045	0.95 (0.88–1.02)	0.133

### 3.4 Urinary MtDNA and association with ccf‐MtDNA in MHD patients

**FIGURE 2:**
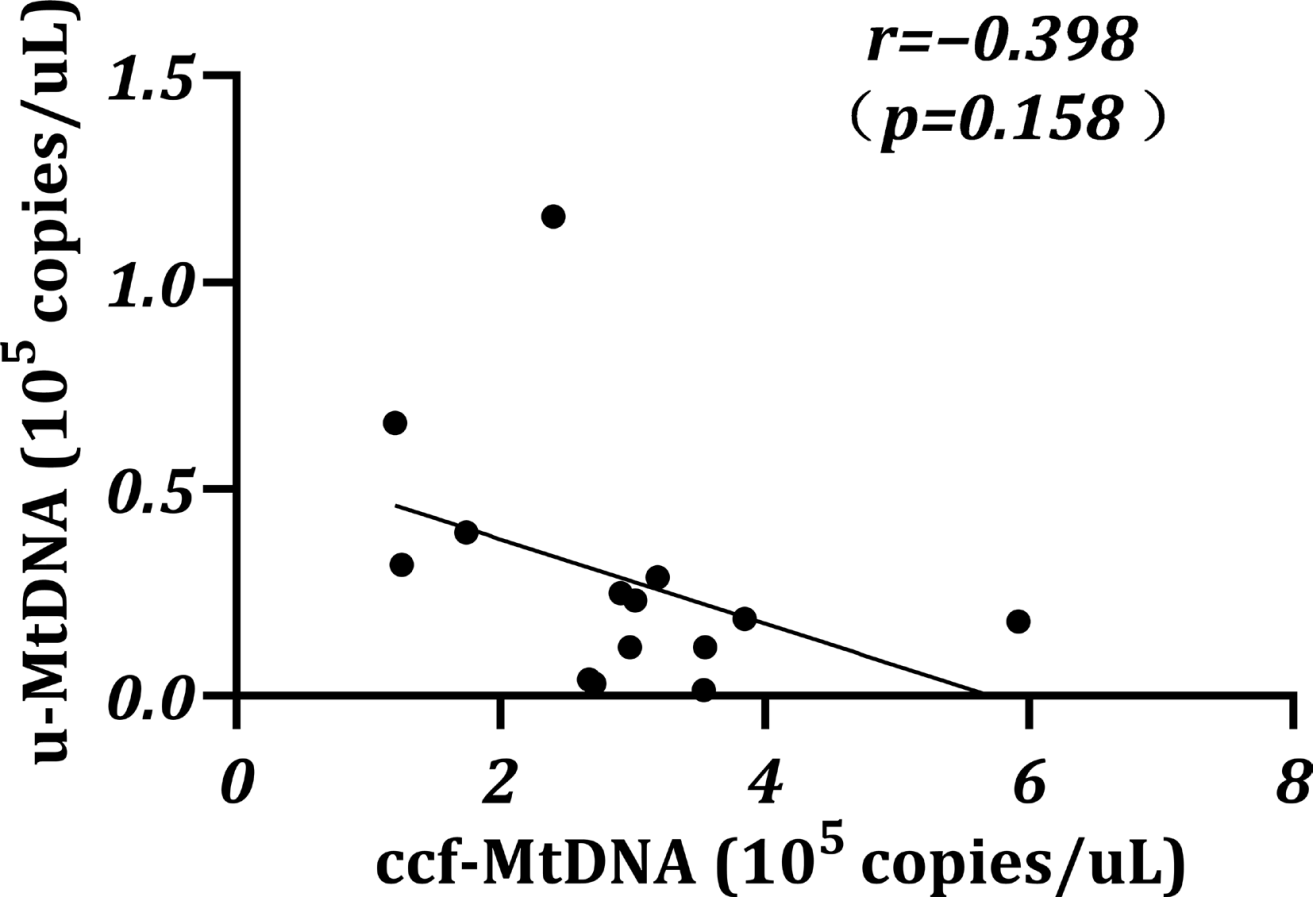
Spearman's correlation between urinary MtDNA and ccf‐MtDNA in patients with residual urine production. The results are represented as scatter plots, where each dot represents data obtained from one subject sample. *r*: Correlation coefficient. *n* = 14. *p < 0.05

In the present study, we confirmed that patients with anuria were more likely to have higher levels of ccf‐MtDNA. This prompted us to investigate whether the excessive ccf‐MtDNA might be partly due to the lack of urinary MtDNA excretion. We measured urinary MtDNA content in patients with residual (*n* = 14) and found that MtDNA was readily detectable in urinary supernatant (median, 0.21 × 10^5^ copies/μl; range, 0.01–1.16 × 10^5^ copies/μl). Meanwhile, although not statistically significant, an inverse correlative trend was observed between urinary MtDNA and ccf‐MtDNA (Figure 2; *r* = −0.398, *p* = 0.158). As previously reported, kidney is responsible for scavenging circulatory MtDNA via glomerular hyperfiltration.^25^ Based on this, it is tempting to speculate that preserving more renal function might be an effective way to eliminate MtDNA in the blood.

### 3.5 Mitochondria appear to be impaired more severely in patients with higher ccf‐MtDNA

**FIGURE 3:**
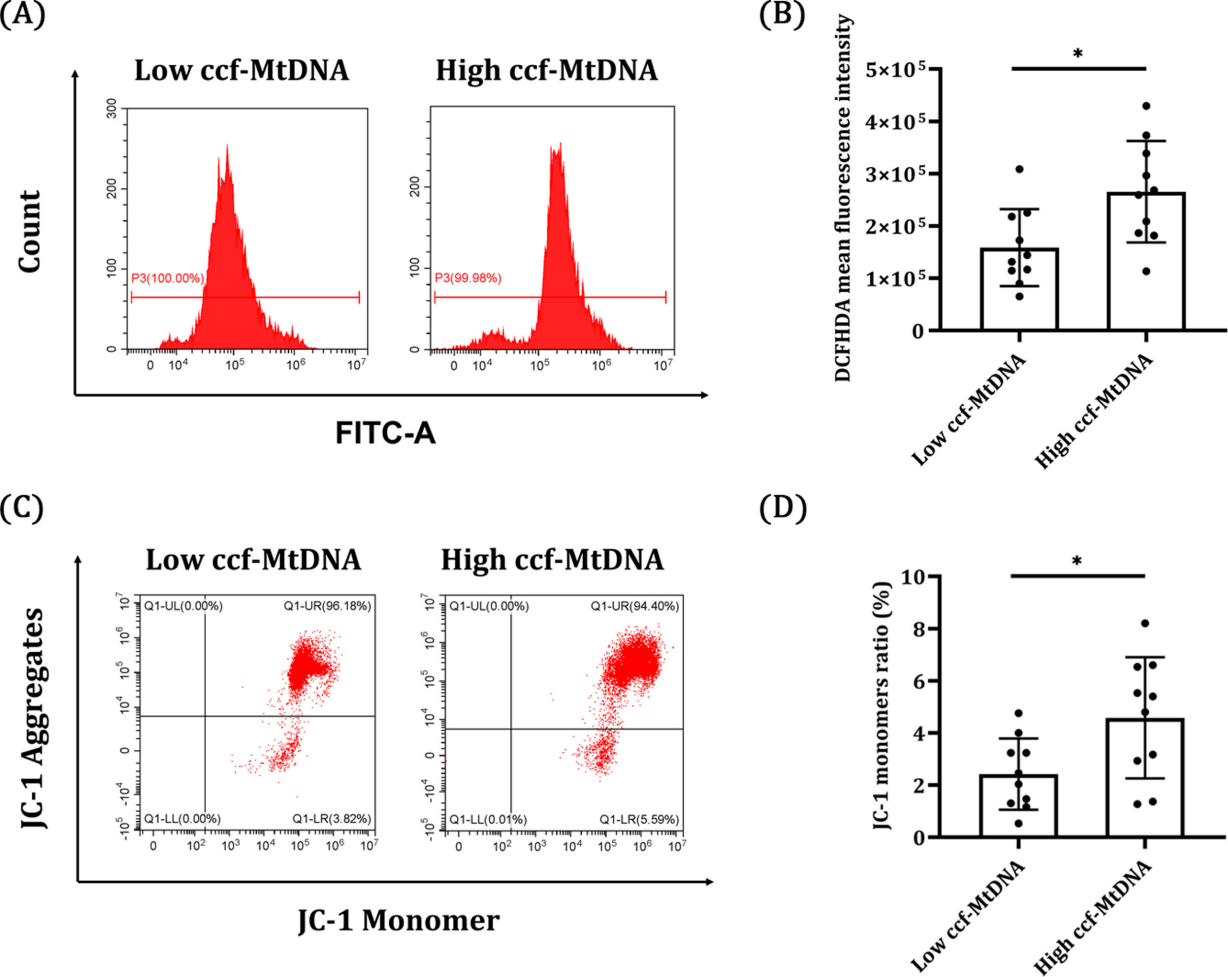
Mitochondria appear to be impaired more severely in patients with higher ccf‐MtDNA. (A, B) ROS generation was analyzed by flow cytometry using DCFH‐DA. (C, D) Loss of Δψm was measured by flow cytometry using the JC‐1 mitochondrial probe. Low ccf‐MtDNA groups, *n* = 10; High ccf‐MtDNA groups, *n* = 10. *p < 0.05

MtDNA is packaged into nucleoids in mitochondria. In order to define the relationship between mitochondrial impairment and MtDNA release, we randomly selected 10 patients from the high and low ccf‐MtDNA MHD groups and measured the ROS and Δψm in PBMCs by DCFH‐DA and JC‐1 staining, respectively. As expected, FACS analysis showed that ROS production was significantly increased, while mitochondrial Δψm was significantly decreased in the high ccf‐MtDNA group (Figure 3; both *p* < 0.05). This finding suggests that mitochondria might be impaired more severely in patients with higher ccf‐MtDNA, even in easily obtainable PBMCs.

## 4 DISCUSSION

The high prevalence of exaggerated systemic inflammation is well established among MHD patients.^26^ However, the mechanisms triggering such response remain to be fully revealed. Recently, ccf‐MtDNA has been in the spotlight as an endogenous danger molecule that can potentially elicit inflammation.^20^ Given this, it would be of interest to investigate whether such relationship between ccf‐MtDNA and inflammation could also be presented in MHD patients. The primary findings of our research were as follows. (1) The “out of place” MtDNA may be involved in the sterile inflammatory response of MHD. (2) A longer dialysis vintage and the presence of anuria predict higher ccf‐MtDNA levels in MHD patients. (3) Kidney may be partly responsible for MtDNA excretion. (4) Mitochondrial impairment likely has a contribution to MtDNA leakage.

Many chronic diseases such as cardiovascular disease, cancer, chronic kidney disease, diabetes, and neurodegenerative diseases, among others, are initiated or worsened by systemic inflammation.^27^, ^28^ Nevertheless, evidence from recent works suggest that in many case, plasma inflammatory cytokines increase with disease progresses independently of antigenic stimulation, such as infections.^29^ Mitochondria are derived from bacteria that were engulfed by the ancestors of today's eukaryotic cells more than a billion years ago and now produce virtually all of the cellular energy.^30^ Due to their bacterial ancestry, mitochondrial‐derived moleculars, such as MtDNA, cardiolipin, and formyl peptides, can act as DAMP agents, triggering the same pathways that respond to pathogen‐associated molecular patterns (PAMPs).^10^ That is, mitochondria not only participate in danger signaling inside the cell but are also a major source of molecule able to activate an innate immune response.

Recently, it has been shown that ccf‐MtDNA can be detected in many pathological conditions which are characterized by a chronic inflammatory status.^7^, ^8^, ^9^, ^10^, ^11^ Similarly, the clinical significance of ccf‐MtDNA in kidney disease has also been reported.^12^, ^13^ Despite that, the possible contribution of this mitochondrial DAMP to the inflammatory milieu that characterizes MHD has not been clarified clearly. In our study, we found that ccf‐MtDNA was significantly elevated in MHD patients, and that IL‐6 and TNF‐α had a positive correlation with ccf‐MtDNA. The positive correlation between cytokines and ccf‐MtDNA is compatible with our hypothesis that circulating MtDNA may act as a mitochondrial DAMP to activate cellular immunity in MHD patients. For MHD patients, two main approaches to decrease inflammatory load were proposed: elimination of factors triggering inflammation or direct removal of inflammatory mediators.^31^ However, to date, it has been still difficult to ameliorate the chronic inflammatory state in MHD patients, especially cytokines such as IL‐6 and TNF‐α, which could not be removed by routine hemodialysis patterns.^32^ Given the known immune‐activating properties of MtDNA, it seems logical to hypothesize that reducing plasma MtDNA might be able to ameliorate the inflammatory status in MHD patients. To achieve this goal, identification of the risk factors and underlying mechanism of its high levels becomes very important.

Multivariable regression analysis proved that a longer dialysis vintage and the presence of anuria were independent risk factors/predictors for higher ccf‐MtDNA levels in MHD patients. For patients under maintenance dialysis, quitting or postponing hemodialysis entails inescapable death. Therefore, retention of renal function seems to be a possible option, as it has been shown to be a predictor of lower ccf‐MtDNA in MHD patients. Indeed, by measuring the urinary MtDNA content in patients with residual urine, we found that although not statistically significant, there was a negative correlative trend between urine MtDNA and ccf‐MtDNA. The results of this study strongly support the idea that kidney is responsible for scavenging plasma MtDNA.^25^, ^33^ Previous studies proposed that circulating MtDNA is originated by passive leakage from broken mitochondria.^7^ Then, to test whether mitochondrial damage exists in MHD subjects, we chose PBMCs as a surrogate biopsy specimen to offer valid information about “general” mitochondrial health and quantified the alternations in ROS and Δψm by flow cytometry. As expected, ROS production was increased and Δψm was decreased in the high ccf‐MtDNA subgroup, indicating that mitochondria were impaired more severely in higher ccf‐MtDNA patients. Thus, it might be hypothesized that the maintenance of healthy mitochondria would assist in reducing MtDNA release.

Our study has several limitations. First, due to the retrospective cross‐sectional study design, a cause‐and‐effect relationship needs to be established in future studies. Second, the study suffers from a multitude of potentially confounding factors. Future research with a large sample size, different patient‐level factors and controlling for confounding factors is needed. Third, as only a trend was observed in our study, the relationship between urinary MtDNA and ccf‐MtDNA requires further analysis before clear conclusions can be drawn. Fourth, we used PBMCs as an alternative cellular source instead of tissue biopsy. More experiments are required to unequivocally determine the source of ccf‐MtDNA.

## 5 CONCLUSIONS

**FIGURE 4:**
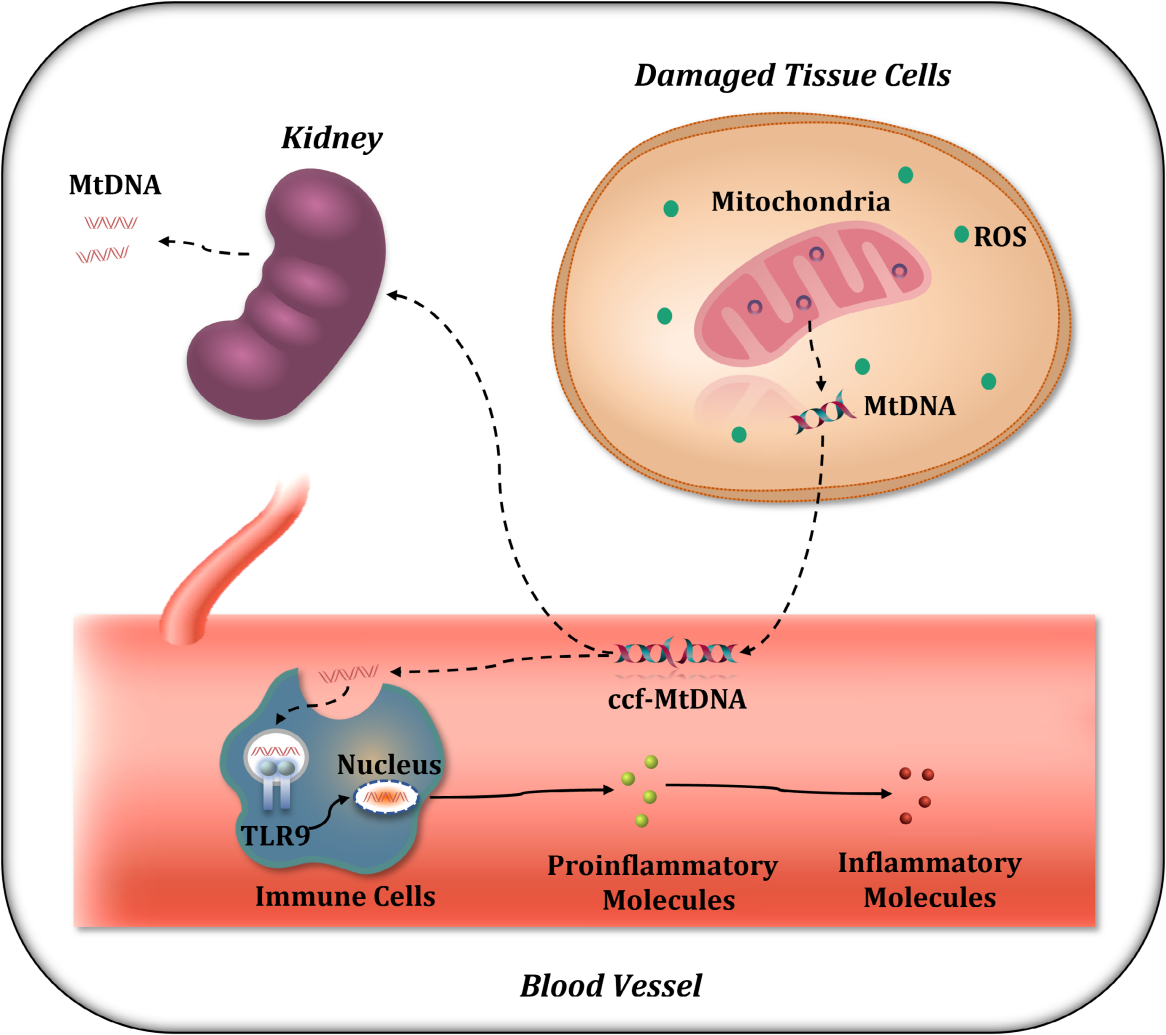
Schematic representation of MtDNA generation and diffusion and its role as a DAMP in inflammation. Mitochondria are impaired in cells in a subset of patients undergoing maintenance hemodialysis, which may lead to the release of MtDNA from damaged mitochondria to the extracellular fluid. Some MtDNAs are excreted into the urinary space, while the MtDNAs remaining in the blood are recognized and combine with PRRs, such as TLR9, in immune cells, leading to the transcriptional activation of pro‐inflammatory cytokines. Pro‐inflammatory cytokines then mature and transform into inflammatory cytokines, amplifying the inflammatory cascade, which ultimately results in inflammatory injuries

In conclusion, we found that ccf‐MtDNA is highly implicated in the immune response, and its level may be causally associated with urinary excretion and mitochondrial damage in MHD patients. A schematic diagram of the proposed pathway is shown in Figure 4. From a future perspective, identification of the role of ccf‐MtDNA in diseases is importance for designing new therapeutic strategies against MtDNA or its receptors to reduce a harmful immune activation.

## CONFLICT OF INTEREST

The authors declare no conflict of interest.

## Data Availability

All the data used to support the findings of this study are included within the article.
